# Exposure to maternal obesity during suckling outweighs *in utero* exposure in programming for post-weaning adiposity and insulin resistance in rats

**DOI:** 10.1038/s41598-019-46518-9

**Published:** 2019-07-12

**Authors:** Grace George, Sally A. V. Draycott, Ronan Muir, Bethan Clifford, Matthew J. Elmes, Simon C. Langley-Evans

**Affiliations:** School of Biosciences, University of Nottingham, Sutton Bonington Campus, Loughborough, Leicestershire LE12 5RD UK

**Keywords:** Obesity, Animal disease models

## Abstract

Exposure to maternal obesity during early development programmes adverse metabolic health in rodent offspring. We assessed the relative contributions of obesity during pregnancy and suckling on metabolic health post-weaning. Wistar rat offspring exposed to control (C) or cafeteria diet (O) during pregnancy were cross-fostered to dams on the same (CC, OO) or alternate diet during suckling (CO, OC) and weaned onto standard chow. Measures of offspring metabolic health included growth, adipose tissue mass, and 12-week glucose and insulin concentrations during an intraperitoneal glucose tolerance test (ipGTT). Exposure to maternal obesity during lactation was a driver for reduced offspring weight post-weaning, higher fasting blood glucose concentrations and greater gonadal adiposity (in females). Males displayed insulin resistance, through slower glucose clearance despite normal circulating insulin and lower mRNA expression of *PIK3R1* and *PIK3CB* in gonadal fat and liver respectively. In contrast, maternal obesity during pregnancy up-regulated the insulin signalling genes *IRS2*, *PIK3CB* and *SREBP1-c* in skeletal muscle and perirenal fat, favouring insulin sensitivity. In conclusion exposure to maternal obesity during lactation programmes offspring adiposity and insulin resistance, overriding exposure to an optimal nutritional environment *in utero*, which cannot be alleviated by a nutritionally balanced post-weaning diet.

## Introduction

Annually, 5 million deaths and $673 billion in global healthcare expenditure are attributed to diabetes, with the prevalence predicted to rise substantially to over 10% of the world’s population by 2040^1^. Type-2 diabetes accounts for the majority of diabetes diagnoses, with the strongest risk factor for the disease being excess body fat^2^. Understanding the mechanisms which lead to the disease is therefore highly important. A hallmark characteristic is loss of function of normal circulating insulin, leading to insulin resistance^3^. Normal regulation of insulin binding to the insulin receptor, activates a series of downstream phosphorylation events, activation of insulin receptor substrate proteins (IRS2), type 1 A phosphatidylinositol 3-kinase (PI3K), and the serine/threonine-protein kinase AKT2, respectively. This leads to reduced hepatic glucose output^4^ and increased glucose uptake in skeletal muscle and adipose tissue^5^, and lipogenesis through downstream activation of sterol regulatory element binding protein 1-c (SREBP-1c)^6^. Dysregulation of critical components of the insulin signalling pathway is associated with the pathogenesis of insulin resistance and hyperglycaemia^7–9^.

Small animal models of maternal obesity have provided robust evidence to suggest that metabolic disease can be programmed by adverse maternal nutrition during early-life, including abnormal glucose homeostasis and insulin signalling in offspring, insulin resistance, and obesity^10–15^. For example, male offspring of dams fed a high-fat diet throughout pregnancy and lactation, manifest reduced adipose tissue expression of the key genes within the insulin signalling pathway including insulin receptor, IRS1, p85α and P110ß (regulatory and catalytic subunits of PI3K) and AKT2^16^, reduced hepatic expression of P85α^17^ and IRS1^18^ and increased AKT expression in skeletal muscle^13,19^. To attempt to tease apart the important critical windows of programming, some studies have used cross-fostering, providing insight in to the effects of exposure during suckling alone on offspring metabolism^18,20,21^. However, high-fat diets often use large quantities of a narrow range of fatty acids and are less valuable as models of maternal obesity that reflect varied human overnutrition^22^.

We have previously discussed the advantages of the cafeteria diet used as a model of maternal obesity in rats, importantly that it more closely reflects maternal obesity in humans^23,24^. Many cafeteria diet protocols are still limited by the scope of variation in highly palatable foods offered to the animals, frequently with a panel of 6–12 foods^23,25,26^. We have developed a more varied protocol, retaining the effect of exposure to novel, palatable items and maximising the obesogenic impact. Using this approach, we demonstrated that cross-fostering was effective in separating the prenatal and postnatal effects of maternal obesity on rat offspring weight and adiposity pre-weaning, with exposure during suckling associated with greater adiposity in 2-week-old male offspring^24^. The aim of this present study was to establish the relative contributions of maternal obesity during pregnancy and lactation on offspring growth, adiposity, glucose tolerance and insulin resistance post-weaning.

## Results

### Effect of maternal diet on offspring weight and body composition

#### Offspring weights

**Figure 1:**
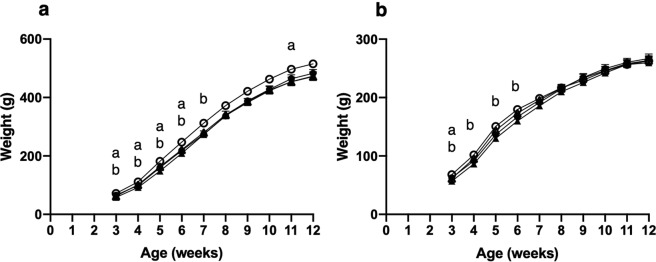
Offspring weight from weaning to 12 weeks of age. Values are for are mean and SEM. (**a**) Male offspring, (**b**) Female offspring. Four groups of cross-fostered offspring were studied: offspring exposed to a chow diet during pregnancy cross-fostered to a chow fed dam during lactation ( CC, *n* 16) or a cafteria fed dam ( CO, *n* 15–16), offspring exposed to a cafeteria diet during pregnancy cross-fostered to a chow fed dam during lactation ( OC, *n* 13–16) or a cafeteria fed dam ( OO, *n* 11–12). (**a**) Effect of maternal pregnancy diet (*P* < 0.05). (**b**) Effect of maternal lactation diet (*P* < 0.05).

The effects of exposure to maternal obesity both during gestation and lactation on male and female offspring growth is demonstrated in Fig. 1. Both stages of maternal dietary exposure were associated with significantly lighter female weights at week 3 and male offspring weights up to week 6 (*P* < 0.05). However, exposure to maternal obesity during lactation was associated with lighter offspring weight, which was more evident in females, with CO and OO offspring demonstrating lighter weights up to week 6 (*P* < 0.05). Offspring from CO, OC, and OO groups demonstrated some evidence of catch up growth thereafter through greater weight gain (*P* < 0.05). Fluctuations were seen in male offspring weights of the OO and OC groups, demonstrated by lighter weights at week eleven, compared to offspring exposed to a control diet during this period (*P* < 0.05). By week 12, there was no significant influence of maternal treatment on offspring weight.

#### Offspring adiposity

Offspring gonadal and perirenal adipose fat pad mass was measured at 4 and 12 weeks of age (Table 1). 4 and 12-week-old male offspring had greater perirenal fat mass than female offspring, whereas female offspring had greater gonadal adiposity (*P* < 0.05). 12-week-old female offspring exposed to maternal obesity during lactation had 26% greater gonadal adiposity than offspring exposed to a control diet during this period (*P* = 0.030), with OO offspring demonstrating almost 30% greater adiposity compared to CC. No differences were seen between other dietary groups or at 4 weeks of age.

**Table 1:** Offspring adiposity.

Gender	Age (weeks)	Group	Perirenal fat (% body weight)		Gonadal fat (% body weight)	
			Mean	SEM	Mean	SEM
Male	4	CC	0.45	0.03	0.41	0.03
		CO	0.54	0.03	0.44	0.03
		OC	0.49	0.05	0.40	0.05
		OO	0.48	0.02	0.44	0.03
	12	CC	2.03	0.18	1.75	0.15
		CO	2.07	0.19	1.76	0.14
		OC	2.06	0.19	1.86	0.16
		OO	2.45	0.19	1.93	0.16
Female	4	CC	0.34	0.02	0.63	0.03
		CO	0.32	0.04	0.57	0.06
		OC	0.39	0.05	0.52	0.05
		OO	0.34	0.03	0.63	0.03
	12	CC	1.10	0.11	2.21	0.21
		CO	1.14	0.07	*2.34	0.25
		OC	1.04	0.14	1.91	0.24
		OO	1.41	0.13	*2.87	0.25

Values are for mean and SEM. Offspring adipose tissue mass of four groups of cross-fostered offspring at 4 or 12 weeks of age: offspring exposed to a chow diet during gestation cross-fostered to a chow fed dam during lactation (CC, *n* 15–16) or a cafteria fed dam (CO, *n* 16), offspring exposed to a cafeteria diet during gestation cross-fostered to a chow fed dam during lactation (OC, *n* 16) or a cafeteria fed dam (OO, *n* 12).

Effect of sex for gonadal and perirenal adiposity at 4 and 12 weeks of age (*P* < 0.05).

*Effect of maternal lactation diet (*P* = 0.030).

### Effect of maternal diet on offspring glucose and insulin concentrations

**Figure 2:**
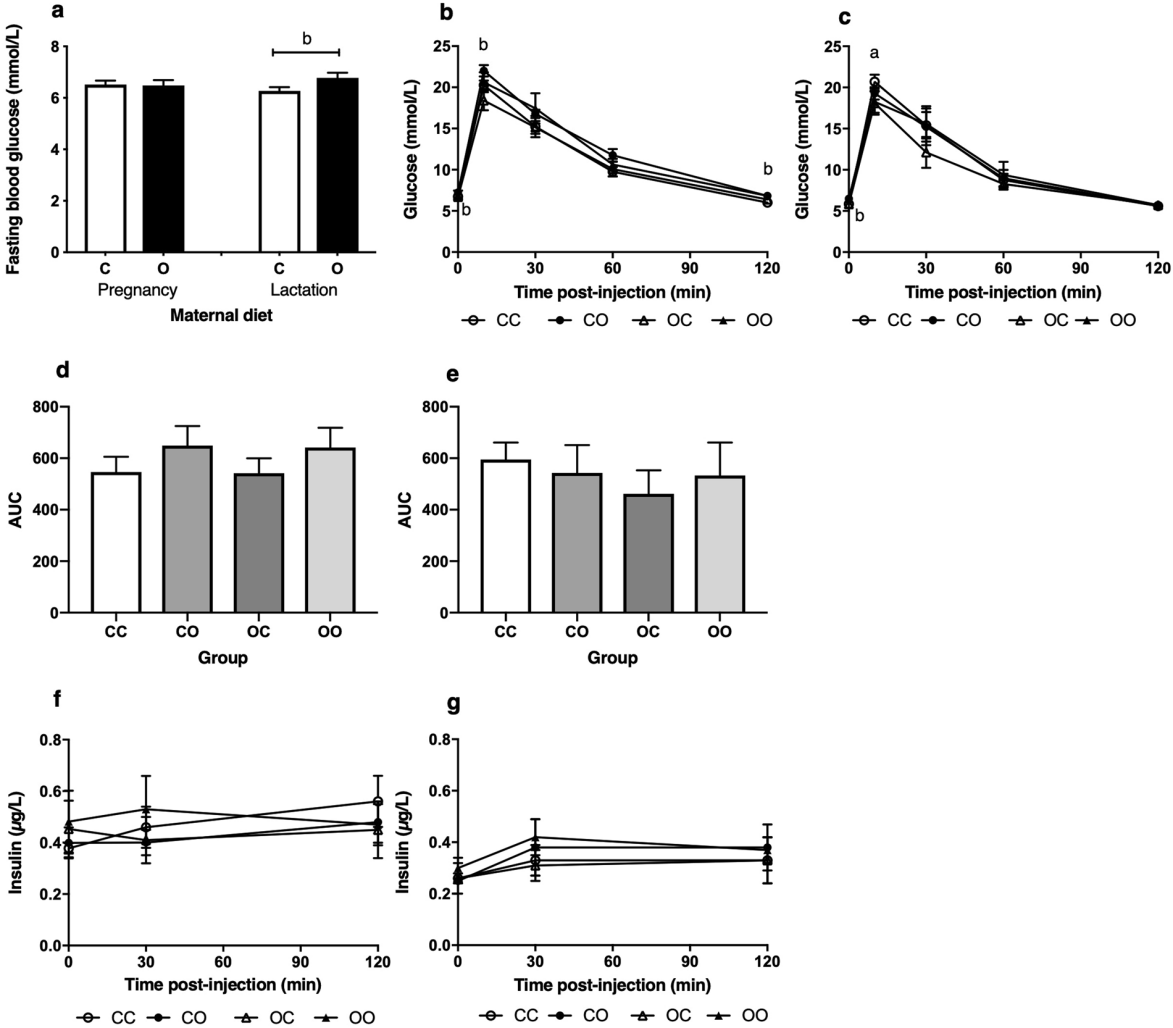
Offspring glucose and insulin concentrations at 12 weeks of age. Values are for mean and SEM. Values are for mean and SEM. (**a**) Fasting blood glucose concentrations at baseline for both male and female offspring combined, exposed to a control diet (C, *n* 20) and cafeteria diet (O, *n* 24) during gestation, or lactation (C *n* 20, O *n* 24). Offspring blood glucose concentrations measured at baseline, 10, 30, 60 and 120 mins post-injection of glucose during an ipGTT, for (**b**) Males (**c**) Females. Offspring ipGTT AUC for (**d**) Males, (**e**) Females. Offspring plasma insulin concentrations for (**f**) Males, (**g**) Females. Four groups of cross-fostered offspring were studied: offspring exposed to a chow diet during pregnancy cross-fostered to a chow fed dam during lactation (CC, *n* 9–14) or a cafeteria fed dam (CO, *n* 10–14), offspring exposed to a cafeteria diet during pregnancy cross-fostered to a chow fed dam during lactation (OC, *n* 10–13) or a cafeteria fed dam (OO, *n* 9–10). Effect of sex for ipGTT glucose concentrations at baseline, 60 minutes and 120 minutes post-injection (*P* < 0.05) and insulin concentrations at baseline and 120 minutes (*P* < 0.05). (a) Effect of maternal pregnancy diet (*P* = 0.024). (b) Effect of maternal lactation diet (*P* < 0.05).

The 12-week-old male and female offspring cross-fostered to dams on a cafeteria fed diet during lactation (CO, OO) had significantly higher fasting blood glucose concentrations compared to offspring exposed to a control diet during this period (*P* = 0.031) (Fig. 2a). Offspring of OC mothers had similar fasting glucose concentrations to CC. Following intraperitoneal administration of glucose, blood glucose reached a peak at 10 minutes post-injection in all groups. A higher peak glucose concentration was observed in male offspring exposed to cafeteria diet during lactation alone (*P* = 0.047) (Fig. 2b). In female offspring exposed to maternal obesity *in utero*, peak glucose was significantly lower than in the CC group (*P* = 0.024) (Fig. 2c). Beyond peak glucose there was no difference in concentrations between female groups, but in males the CO and OO groups had elevated glucose through to 120 minutes. All groups returned to similar basal values at two hours but glucose concentrations in male CO and OO reflected that of baseline, with 10% higher concentrations compared to offspring exposed to a control diet during suckling (*P* = 0.040). Although CO and OO male offspring demonstrated elevated areas under the glucose curves (AUC), these differences did not achieve statistical significance (Fig. 2d,e).

There were no significant differences in plasma insulin concentrations between groups of offspring (Fig. 2f,g). Male offspring demonstrated elevated insulin concentrations compared to females, particularly at baseline and two hours (*P* < 0.05). As only male offspring exposed to maternal obesity during lactation demonstrated an altered glucose tolerance, measurements of insulin signalling mRNA expression focused on male rather than female tissues.

### Effect of maternal obesity on insulin signalling mRNA expression in male offspring

#### Liver

**Figure 3:**
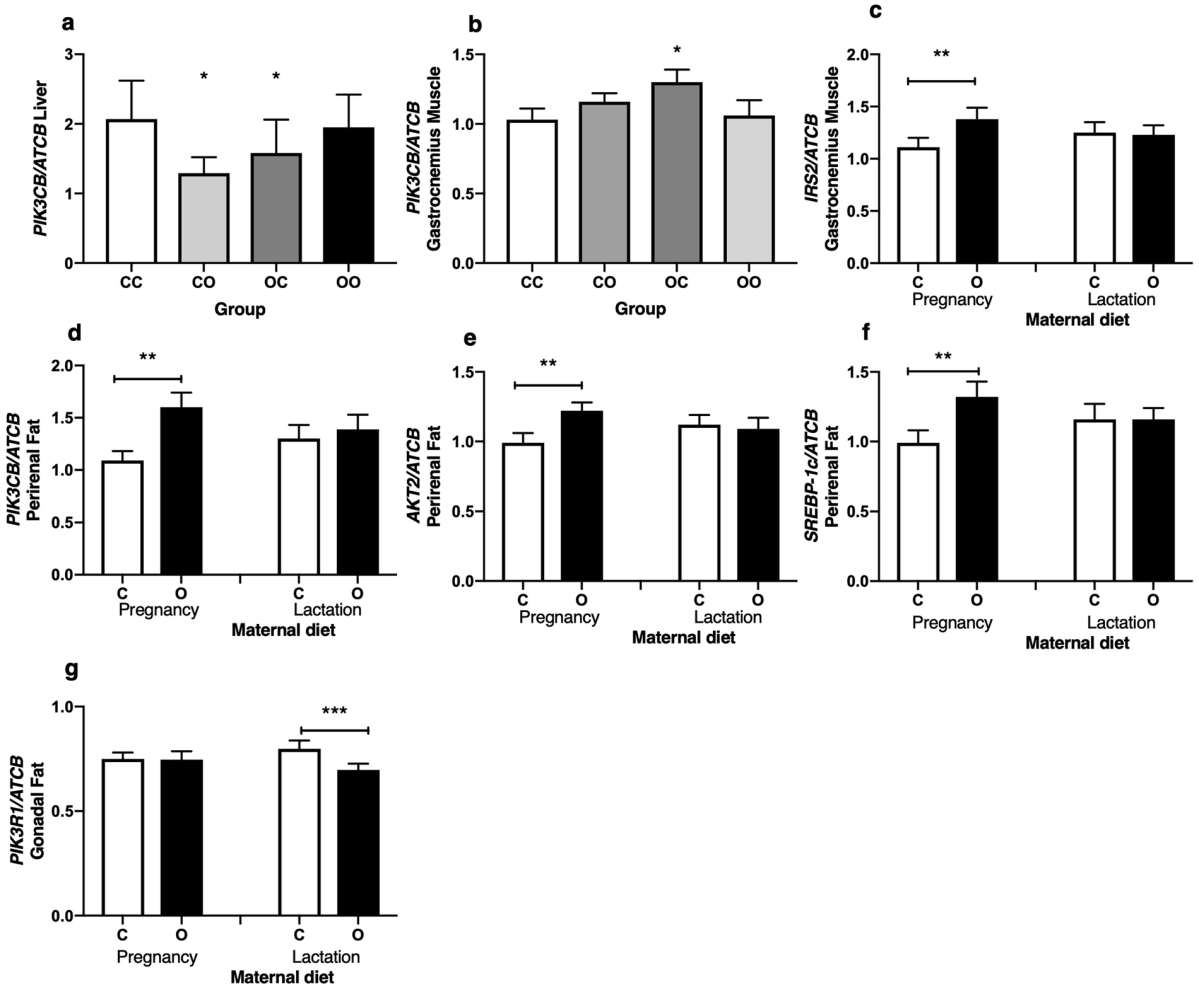
Expression of mRNA for insulin signalling pathway target genes. Values are for mean and SEM. (**a**) Liver mRNA expression of *PIK3CB* at 4 and 12 weeks (combined), (CC, *n* 16), (CO, *n* 15), (OC, *n* 16), (OO, *n* 11). (**b**) Gastrocnemius muscle mRNA expression of *PIK3CB* at 12 weeks of age, (CC, *n* 7), (CO, *n* 8), (OC, *n* 5), (OO, *n* 5). (**c**) Gastrocnemius muscle mRNA expression of *IRS2* at 4 and 12 weeks (combined), comparing offspring exposed to a control diet (C, *n* 26) or cafeteria diet (O, *n* 21) during pregnancy or lactation (C *n* 23, O *n* 24). Perirenal adipose tissue mRNA expression of (**d**) *PIK3CB* and (**e**) *AKT2* at 4 weeks of age, comparing offspring exposed to a control diet (C, *n* 14–15) or cafeteria diet (O, *n* 10–11) during pregnancy, or lactation (C *n* 12–14, O *n* 11–13). (**f**) Perirenal adipose tissue mRNA expression *SREBP-1c* at 4 and 12 weeks (combined), comparing offspring exposed to a control diet (C, *n* 25) or cafeteria diet (O, *n* 21) during pregnancy, or lactation (C *n* 24, O *n* 22). (**g**) Gonadal adipose tissue mRNA expression of *PIK3R1* at 4 and 12 weeks (combined), comparing offspring exposed to a control diet (C, *n* 28) or cafeteria diet (O, *n* 27) during pregnancy, or lactation (C *n* 30, O *n* 25). mRNA expression was normalised to the housekeeping gene *ATCB*. *Effect of maternal pregnancy diet and maternal lactation diet (*P* < 0.05). **Effect of maternal pregnancy diet (*P* < 0.05). ***Effect of maternal lactation diet (*P* = 0.049).

Liver is an important tissue involved in the regulation of glucose homeostasis, therefore the mRNA expression of a panel of genes was measured to investigate if maternal diet during pregnancy or lactation disturbed this process. Liver mRNA expression was measured in male offspring at 4 and 12 weeks of age. Supplementary Table S1 highlights that there were no significant mRNA expression changes between groups when analysed in 4 and 12-week-old male offspring separately. To smooth out effects of variation in expression across the two different ages and focus on effects of maternal diet, we performed further analysis of mRNA expression in the combined age groups. This greater powered analysis demonstrated an interaction of maternal pregnancy and lactation diet associated with overall lower *PIK3CB* mRNA expression in CO and OC male offspring by 37% and 23% respectively (Fig. 3a) (P = 0.022). 4-week-old offspring demonstrated greater mRNA expression of *IRS2*, *PIK3CB* and *PIK3R1* than other age groups (*P* < 0.05).

#### Gastrocnemius muscle

Skeletal muscle was also an important tissue to measure insulin signalling mRNA expression given its key role in glucose uptake. An interaction of maternal pregnancy diet and maternal lactation diet demonstrated 12-week-old OC male offspring had 26% greater *PIK3CB* mRNA expression in gastrocnemius muscle than CC (*P* = 0.034; Fig. 3b), which was not observed at 4 weeks. Figure 3c demonstrates that *IRS2* mRNA expression was 24% higher in male offspring exposed to maternal obesity during pregnancy across both age groups compared to offspring exposed to a control diet (*P* = 0.043). 4-week-old offspring demonstrated greater mRNA expression of *IRS2* and *PIK3R1* than at 12 weeks (*P* < 0.05). No differences were seen between dietary groups for the other target genes at 4 and 12 weeks (Supplementary Table S2).

#### Perirenal adipose tissue

Although white adipose tissue has a smaller role in glucose uptake than skeletal muscle, visceral adipose tissue is particularly important in the pathogenesis of insulin resistance, including the gonadal and perirenal depots. At 4 weeks, *PIK3CB* (Fig. 3d) and *AKT2* (Fig. 3e) mRNA expression was higher by 47% and 23% respectively in perirenal adipose tissue of male offspring exposed to maternal obesity during pregnancy compared to offspring exposed to a control diet (*P* < 0.05). These effects were not seen in 12-week-old male offspring. *SREBP-1c* mRNA expression was 33% higher in offspring exposed to maternal obesity before birth across both age groups, compared to offspring exposed to a control diet during this period (*P* = 0.017; Fig. 3f), which was also greater by 41% with analysis of 4-week-old offspring (*P* = 0.016; Supplementary Table S3). 4-week-old offspring demonstrated greater mRNA expression of *IRS2*, *AKT2*, *PIK3CB* and *PIK3R1* than at 12 weeks of age (*P* < 0.05). No differences were seen between dietary groups for the other target genes at 4 and 12 weeks (Supplementary Table S3).

#### Gonadal adipose tissue

Whilst perirenal adipose tissue signalling demonstrated an increase in insulin signalling mRNA expression associated with exposure to maternal obesity during gestation, gonadal adipose tissue demonstrated the opposite effect, highlighting a difference between the two depots. Supplementary Table S4 highlights that there were no significant mRNA expression changes between groups when analysed in 4 and 12-week-old male offspring separately. Analysis combining both age groups (Fig. 3g), demonstrated that offspring exposed to maternal obesity during lactation had 12% lower *PIK3R1* mRNA expression than offspring exposed to a control diet (*P* = 0.049). Although not significant, there was a trend of reduced *AKT2* mRNA expression in 4-week-old offspring (*P* = 0.056), and reduced *IRS2* mRNA expression in 12-week-old offspring (*P* = 0.052), which was associated with maternal obesity exposure during lactation (Supplementary Table S4). 4-week-old offspring demonstrated greater mRNA expression of members of the insulin signalling pathway, except *PIK3R1*, than 12-week-old offspring (*P* < 0.05).

## Discussion

This study utilised a novel cafeteria-feeding regimen previously developed by the authors to maximise maternal obesity to assess the relative effects of maternal overfeeding during pregnancy and lactation on offspring growth, adiposity and metabolic function^23,24^. Using this approach, we identified that a highly varied, true cafeteria diet, comprising of 40 different food items, induced maternal obesity in female rats and exposure to maternal obesity during pregnancy was associated with reduced fetal growth, whereas exposure during lactation was associated with greater male offspring adiposity at 2 weeks of age^24^. Consistent with evidence from previous cross-fostering studies, these current findings show that exposure to maternal obesity during lactation was the main driver for significantly lighter weights from weaning^27^ and adverse metabolic function in offspring^18,20,21^. The findings also confirm that male offspring appear more susceptible to insulin resistance^23,28^ and females to adiposity in adulthood^26,29^. However, the present study was important because it assessed the effect of exposure to maternal obesity, induced through a highly varied cafeteria diet, rather than diets high in specific fatty acids or sugars, during critical windows of development.

Only postnatal exposure to maternal obesity led to adiposity and insulin resistance, and was the main driver for reduced weights post-weaning, suggesting that the milk composition of the dams is key to these programming effects, which are independent of *in utero* exposure. It cannot be ruled out that the lower protein composition of the maternal cafeteria diet contributed to the offspring phenotypes observed but it appears to have no effect on offspring stomach protein content or milk composition at birth^30^. Instead, higher milk concentrations of a range of nutrients are believed to influence offspring development^30,31^. This includes milk concentrations of the satiety adipokine leptin^32^, an important mediator of peripheral insulin sensitivity^33^. This warrants future investigation. Previous research provides some evidence to suggest maternal obesity, rather than the cafeteria diet itself, can exert independent programming effects on offspring growth and metabolism^23,34^. However, cross fostering to separate effects of maternal obesity and cafeteria diet on the phenotype of the offspring in the current study has shown it is unlikely that maternal obesity alone programmes postnatal growth and development.

Smaller offspring, particularly females, caught up in weight in the later stages of life. This was evidenced by increased weight gain and lack of significance between groups. However, growth restriction in early-life followed by catch-up growth is associated with obesity and insulin resistance^29,35–37^. Female offspring exposed to maternal obesity during lactation did display evidence of greater gonadal adiposity in adulthood. We have previously reported that 2 week-old male offspring exposed to maternal obesity during lactation alone demonstrated elevated perirenal adiposity^24^, but the present findings highlight this was lost by adulthood. This early transient visceral obesity phenotype has also been demonstrated in previous research^20,27^. These findings suggest that weaning onto a nutritionally balanced chow diet may alleviate the programming effects of reduced growth, and fat deposition in males but not females. Previous research also suggests that female offspring may be further susceptible to diet-induced obesity^26,38^. Further research would need to identify why these depot and sex-specific programming effects occur, such as the interaction of adverse maternal nutrition and offspring sex-hormones as well as the epigenome^39–41^. The mechanisms of elevated adiposity may be due to programming of adipocyte size, development, and pre-adipocyte differentiation^38,42^ with altered gene expression mediating these effects^27^.

To the best of our knowledge, this is the only study to investigate glucose handling using the cafeteria diet in combination with cross fostering. As a consequence, glucose rather than insulin tolerance tests were undertaken to gain a physiological overview of any changes in glucose homeostasis resulting from exposure to cafeteria diet, rather than identification of the detailed mechanisms behind any resulting phenotype. At 12 weeks of age, both male and female offspring exposed to maternal obesity during lactation demonstrated elevated fasting blood glucose levels compared to offspring exposed to a control diet. Although there is no classification in rats, this would indicate impaired fasting glucose (IFG) in humans^43^. However, offspring did not demonstrate impaired glucose tolerance (IGT), defined in humans as raised blood glucose levels, two-hours following an oral GTT^43^. Glucose concentrations two-hours post-injection were below baseline values, which may be because animals were fasted overnight, as this can enhance insulin stimulated glucose uptake^44,45^. This indicates whilst offspring demonstrated hyperglycaemia and a pre-diabetic state, they did not appear clinically glucose intolerant. Male offspring exposed to maternal obesity during lactation did appear more susceptible to altered glucose homeostasis demonstrated by significantly greater peak glucose levels and slower clearance of the glucose load compared to offspring exposed to a control diet. However, no changes in insulin concentrations were observed between groups.

There were only minor fluctuations in insulin concentrations throughout the time-course studied, but a peak in plasma insulin levels would usually be observed^46^. Therefore, for future experiments extra time-points could be considered. However, in the early-stages of insulin resistance, blood glucose concentrations are elevated despite normal circulating insulin concentrations^5^, which is due to a suboptimal biological response to insulin and failure to transmit the insulin signal^8^. This suggests that male offspring exposed to maternal obesity during lactation displayed early signs of insulin resistance. Further investigation was carried out to establish the potential mechanisms of insulin resistance in male offspring, through the investigation of mRNA expression of key insulin signalling genes within the target tissues, that is liver, skeletal muscle and adipose tissue. There were clear differences between exposure to maternal obesity before birth and during lactation, with a gene expression profile favouring insulin resistance in offspring exposed to maternal obesity during lactation.

*PIK3R1* mRNA expression was lower in gonadal adipose tissue of male offspring exposed to maternal obesity during lactation across the lifespan. *PIK3R1* and *PIK3CB* genes code for the p85α regulatory and p110ß catalytic subunits of *PI3K* respectively, an important mediator of insulin signalling which activates downstream targets of the pathway, including *AKT2*, leading to glucose uptake^47^. Lower p85α protein expression in adipose tissue is associated with maternal high-fat feeding^16^, and maternal undernutrition during early-life^48^, suggesting it is a particular target for programming by adverse maternal nutrition. Male offspring exposed to maternal obesity during suckling also displayed a minor effect of lower gonadal mRNA expression of *AKT2* at 4 weeks, and *IRS2* at 12 weeks, an upstream activator of PI3K. Whilst white adipose tissue only accounts for a small amount of glucose uptake, it is susceptible to insulin resistance due to adverse maternal nutrition in offspring^16,49^. One limitation of the current study was that we only considered mRNA and not protein expression. If verified at the protein level the observed changes in insulin signalling may partly explain greater glucose concentrations in this group offspring.

Male CO and OC offspring displayed signs of hepatic insulin resistance, through lower mRNA expression of *PIK3CB* across the post-weaning lifespan. For OC offspring, this may be evidence of metabolic disturbance and abnormal glucose homeostasis may be present later in life^50^. Whereas CO offspring demonstrated a greater glucose response than other groups, suggesting the hepatic insulin resistance could be additive to the gonadal insulin resistance. In skeletal muscle and perirenal adipose tissue, cross-fostering highlighted the opposite effect in offspring exposed to maternal obesity during gestation, suggesting insulin sensitivity. In skeletal muscle, this was associated with greater mRNA expression of *PIK3CB* in OC offspring at 12 weeks and of *IRS2* in OC and OO offspring across the lifespan. This may explain why male OC offspring demonstrated similar baseline blood glucose concentrations and AUC to CC, as well as lower peak concentrations, given that skeletal muscle is the major tissue involved in glucose uptake. Maternal obesity during gestation was also associated with greater perirenal adipose tissue mRNA expression of *AKT2*, and *PIK3CB* at 4 weeks, but these effects were lost at 12 weeks, whereas *SREBP-1c* was greater across the lifespan. This corresponds with findings that showed male offspring exposed to high-fat feeding before birth had elevated expression of *SREBP-1c* and the enhanced insulin signalling, thought to drive lipogenesis^51^. This suggests a profile favouring enhanced adiposity in male offspring exposed to maternal obesity *in utero*, which although was not seen by 12 weeks, may be seen later in life. In addition, early-insulin sensitivity could give rise to insulin resistance later in life, as seen in offspring exposed to low protein diets *in utero* who tend to demonstrate insulin resistance after one year of age^50,52^. Further study would be required to assess if hyperinsulinemia and insulin resistance was observed in older offspring. This research has highlighted a difference in response between the two visceral fat depots, supporting previous studies that suggest they are programmed differently dependent on the maternal nutritional insult and its timing^39,53^. Further research is required to understand why these programming mechanisms occur, such as the potential for epigenetic modifications throughout the lifespan^16,41,54^. However, our previous studies have suggested a limited role for DNA methylation in determining gene expression in offspring of cafeteria diet fed rats^55^.

In summary, these findings highlight that exposure to maternal obesity during lactation can override genetics and *in utero* exposure to a normal maternal environment in programming for offspring obesity in females and insulin resistance across the lifespan in males, which cannot be alleviated by weaning onto a nutritionally balanced post-weaning diet. In contrast, exposure to maternal obesity during pregnancy was associated with insulin sensitivity in male offspring. This would warrant future investigation to understand the mechanisms behind the observed phenotypes and if altered insulin signalling is also seen in females, as well as the role of the epigenome. Whilst caution should be made in extrapolating these findings to humans, understanding the metabolic mechanisms of obesity and insulin resistance offers the potential target for nutritional intervention during breastfeeding.

## Methods

### Animal procedures

Animal procedures including maternal feeding and cross-fostering have been previously reported^24^. All animal procedures were performed in accordance with the Animals (Scientific Procedures) Act 1986 under Home Office licence and were approved by the Animal Ethics Committee of the University of Nottingham, UK. Briefly, animals were subjected to a controlled 12-hour light, 12-hour dark cycle, in conditions at 20–22 °C and 45% humidity. They were housed in plastic cages with wood-shavings and environmental stimuli and had *ad libitum* access to food and water. Virgin female Wistar rats (Charles River, UK), approximately 4-weeks-old (approximately 95 g; *n* 32) were randomly allocated to be fed either a control chow diet (C; *n* 16) (Teklad Global 18% Rodent Diet, Harlan, Belton, Now Envigo) or a cafeteria diet (O; *n* 16). The cafeteria diet consisted of a range of 40 highly palatable, energy-rich human foods, accompanying a chow diet. Animals were fed their respective diets for 8 weeks prior to mating through to weaning of offspring. At mating, all rats were housed with Wistar stud males and mating confirmed by the appearance of a semen plug. All offspring were cross-fostered within 96 hours of birth. Four groups of cross-fostered offspring were generated: offspring exposed to a chow diet during pregnancy cross-fostered to a chow-fed dam during lactation (CC; *n* 64) or a cafeteria-fed dam (CO; *n* 64), offspring exposed to a cafeteria diet during pregnancy cross-fostered to a chow-fed dam during lactation (OC; *n* 64) or a cafeteria-fed dam (OO; *n* 48). 3-week-old offspring were then weaned onto a chow diet and group housed with rats of the same sex. Offspring were weighed weekly (+/−3 days). Data in this paper focuses only on the outcomes for offspring aged 3–12 weeks. Data on the pregnant and lactating dams and the pre-weaning offspring are presented elsewhere^24^. Importantly, the dams to the offspring presented in this study consumed considerably more energy, fat, total carbohydrate and sugar and were heavier throughout pregnancy and lactation when fed the cafeteria diet. Relative to the control diet-fed dams adipose tissue mass was markedly greater, indicating marked obesity.

### Intraperitoneal glucose tolerance test

At 12 weeks of age (+/−3 days), one male and one female per litter were fasted for approximately 16 hours overnight before determination of glucose tolerance using an intraperitoneal glucose tolerance test (ipGTT). The fasted state is argued to be advantageous for measuring glucose utilization^44,56^. Anaesthetic cream was applied to the tail 30 minutes before a baseline fasting blood glucose concentration was obtained by collecting a small blood sample from the superficial tail vein by removing a small section away from the tail tip using a blade. After 5 minutes, a glucose solution (glucose and normal saline) was administered via an intraperitoneal injection (overall dose of 2 g glucose/kg body weight). At 10, 30, 60, and 120 minutes post-glucose administration, blood was sampled by removing the scab from the tail tip and blood glucose concentration was determined instantly using a SD Codefree Blood Glucose Monitor and testing strips (SD Biosensor). Offspring were then culled, and tissue and plasma collected. Blood samples were collected in duplicate at each time point.

### Tissue and plasma collection

Animals aged 4 or 12 weeks were culled using CO_2_ asphyxia and cervical dislocation. Blood was collected in 1.3 mL EDTA tubes (Sarstedt, Germany) during the ipGTT and post-cull using cardiac puncture, and was stored on ice until plasma could be separated on the same day by centrifugation for 10 min at 14,000 rpm. Plasma was removed from haematocrit using a pipette and stored in 1.5 mL tubes at −80 °C. Liver, gastrocnemius muscle, perirenal and gonadal adipose tissue was collected at 4 weeks of age (+/−3 days), from one male and one female (where possible). Tissue was collected from all remaining offspring at 12 weeks of age (+/−3 days). Gonadal and perirenal adipose tissue was weighed for analysis of adiposity. All tissue collected throughout the study was stored in 1.5 mL tubes snap frozen in liquid nitrogen before being stored at −80 °C. In preparation for RNA extraction, frozen tissue was then crushed in liquid nitrogen.

### Insulin enzyme-linked immunosorbent assay (ELISA)

Plasma samples from baseline, 30 minutes and 120 minutes during the ipGTT were used to measure insulin concentration with the Rat Insulin ELISA (Mercodia AB, Sweden) as per manufacturer’s protocol. The absorbance was read at 450 nm using a Flurostar Optima (BMG Labtech, Germany). The concentration of the unknown samples was then calculated from the mean absorbance of the calibrators using as second order polynomial (quadratic) non-linear standard curve.

### Determination of mRNA expression in liver, gastrocnemius muscle, and adipose tissue

Male offspring aged 4 and 12 weeks were only used for these analyses as females did not exhibit any major differences in ipGTT response. Only offspring that did not undergo ipGTT at 12 weeks were used to measure insulin signalling in the fed state as it is the preferred method to measure insulin action^44,56^. RNA was extracted from 20 mg of liver per sample using the High Pure RNA Tissue Kit (Roche Diagnostics Ltd, UK) as per the manufacturer’s protocol. RNA was extracted from 100 mg adipose tissue per sample, from the gonadal and perirenal fat depots, using the method as previously described^57^. RNA was extracted from gastrocnemius muscle using the RNeasy Fibrous Tissue Mini Kit (Qiagen, UK), with the following modifications: 30 mg of skeletal muscle (frozen and crushed) was added into a 2 mL MagNA Lyser green bead tube (Roche Diagnostics Ltd, UK) with 350 µL Buffer RLT. Tubes were vortexed for 10 seconds and then placed immediately in a MagNA Lyser (Roche Diagnostics Ltd, UK) at 6500 rpm for 40 seconds. Tubes were then centrifuged for 3 minutes at 8000 rpm and at this point the standard manufacturer’s protocol applied. RNA concentrations were then diluted to 50 ng/µL by adding the correct amount of RNase-free water. Samples were then stored at −80 °C until required for cDNA synthesis. The method of determination of RNA concentration and quality, as well as cDNA synthesis has been previously described^57^.

Insulin signalling target genes include: insulin receptor (*INSR*) and downstream components *IRS2*, *PIK3R1* and *PIK3CB* (that code for the respective regulatory p85α subunit and catalytic P110ß subunit of PI3K), *AKT2* and *SREBP-1c and were targeted because of their known differential expression within established models of developmental programming*^13,23,50,55^. Primers were designed using a method previously reported^23^. Primer sequences were published elsewhere for *AKT2*^23^, *INSR*^55^ *IRS2* and *SREBP-1c*^50^. Primer sequences can be seen in Table 2. Forward and reverse primers were ordered dried (0.025 µM synthesis scale, desalt purification) (Sigma-Aldrich, UK) and were prepared as per manufacturer’s instructions and were stored at −20 °C. Real-time PCR was performed using a Light-Cycler 480 (Roche Diagnostics Ltd, UK) and SYBR green (Roche Diagnostics Ltd, UK), as previously described^23,57^. Expression of the genes of interest was normalised to the housekeeping gene *ATCB*, which was unaltered in response to the dietary treatments.

**Table 2:** Primer sequences used for determination of mRNA expression.

Gene name	Protein coding	Forward primer sequence (5′ to 3′)	Reverse primer sequence (5′ to 3′).	NCBI Reference Sequence
*AKT2*	AKT2	CAGAGAGCCGAGTCCTACAGAATAC	GTCATGGGTCTGGAAGGCATA	NM_017093.1
*INSR*	Insulin receptor	GGATTATTGTCTCAAAGGGCTGAA	CGTCATACTCACTCTGATTGTGCTT	NM_017071.2
*IRS2*	Insulin receptor substrate 2	TGAGACCAAGTGGCATCGTT	CTCTTGGGCTCAGTGGGTAGA	NM_001168633.1
*SREBP-1C*	Sterol regulatory element-binding protein-1c	GGAGCCATGGATTGCACATT	AGGAAGGCTTCCAGAGAGGA	NM_053445.2
*PIK3R1*	PI3K subunit P85α	TCTGGCCGAGCAGTTTGC	TTCCAGTCCTTTCTTCTCAATGG	NM_013005.1
*PIK3CB*	PI3K subunit P110ß	TTGCGGCAGGACATGCT	GCCAGAGCGATCTCCTGTTG	NM_053481.2
*ATCB*	ß-actin	CGTGAAAAGATGACCCAGA	CACAGCCTGGATGGCTACGT	NM_031144.3

### Statistical analysis

All data was analysed using Statistical Package for Social Sciences (version 22; SPSS, Inc., Chicago, IL, USA). All data was checked for normal distribution using the Shapiro-Wilk test of normality and normality plots. If data was not normally distributed, data was transformed using the appropriate method based on the skewness of the data. For analysis where duplicate or triplicate results were recorded, firstly the mean of the replicates was calculated. Area under the glucose curve was calculated for intraperitoneal glucose tolerance test data using GraphPad Prism (version 6.00 for Windows, GraphPad Software, La Jolla, California, USA). For the effect of maternal diet on offspring weight, this was assessed separately for males and females using a general linear model two-way ANOVA (fixed factors were maternal pregnancy diet and maternal lactation diet). For the effect of maternal diet on offspring body composition, glucose tolerance and plasma insulin, a general linear model three-way ANOVA was used (fixed factors were maternal pregnancy diet, maternal lactation diet and sex). Two-way ANOVA was then used for males and females separately to highlight any specific sex effects (fixed factors were maternal diet at both stages). To determine the effect of maternal diet on male offspring mRNA expression for each gene of interest and each tissue type across all age groups, a three-way ANOVA was used (fixed factors were age, maternal pregnancy diet, and maternal lactation diet). Two-way ANOVA was used to highlight any specific age effects (fixed factors were maternal diet at both stages). For offspring weights and 12-week-old offspring adiposity, where there was more than one offspring per litter, means per litter were identified and then statistical analysis was performed with this data. *P* < 0.05 was considered statistically significant. Variable *n* numbers presented in the results, mean that some animals were excluded from some analyses due to outliers or data not obtained.

### Ethical approval and informed consent

This study was approved by the Animal Ethics Committee of the University of Nottingham, UK. All animal procedures were performed in accordance with the Animals (Scientific Procedures) Act 1986 under Home Office licence.

## Supplementary information

Supplementary Dataset 1

## Data Availability

The datasets generated during and/or analysed during the current study are available from the corresponding author.
