# Supplementary material for: Exposure to maternal obesity during suckling outweighs in utero exposure in programming for post-weaning adiposity and insulin resistance in rats
Source: Sci Rep. 2019 Jul 12;9:10134. doi: 10.1038/s41598-019-46518-9 (PMC6626015; doi:10.1038/s41598-019-46518-9)
Supplement: Supplementary file 1 — Supplementary Dataset 1 [file 41598_2019_46518_MOESM1_ESM.pdf]

Exposure to maternal obesity during suckling outweighs *in utero* exposure in programming for post-weaning adiposity and insulin resistance in rats.

Grace George, Sally A.V. Draycott, Ronan Muir, Bethan Clifford, Matthew J. Elmes, Simon C. Langley-Evans.

Supplementary Material

Age	Group							
	CC		CO		OC		OO	
	Mean	SEM	Mean	SEM	Mean	SEM	Mean	SEM
**4 weeks**								
INSR/ATCB	1.29	0.17	1.09	0.27	1.24	0.24	1.10	0.17
IRS2/ATCB	3.98	1.06	3.09	0.92	2.96	0.92	3.85	1.64
PIK3R1/ATCB	1.90	0.47	2.12	0.72	1.46	0.43	1.89	0.78
PIK3CB/ATCB	3.11	0.99	1.58	0.40	2.19	0.87	2.95	0.84
AKT2/ATCB	1.45	0.18	1.35	0.13	1.50	0.28	1.30	0.31
SREBP-1C/ATCB	3.34	0.71	2.02	0.57	2.98	0.68	3.21	0.54
**12 weeks**								
INSR/ATCB	1.30	0.11	1.18	0.07	1.15	0.07	1.19	0.07
IRS2/ATCB	1.25	0.14	1.32	0.15	1.06	0.18	1.08	0.16
PIK3R1/ATCB	1.10	0.14	1.06	0.18	0.87	0.07	0.89	0.15
PIK3CB/ATCB	1.03	0.06	0.96	0.11	0.98	0.06	1.12	0.14
AKT2/ATCB	1.22	0.07	1.24	0.12	1.28	0.11	1.16	0.15
SREBP-1C/ATCB	2.60	0.28	2.52	0.39	2.59	0.30	2.52	0.48

Supplementary Table S1: Liver insulin signalling mRNA expression in male offspring.
Values are for mean and SEM. Male offspring insulin signalling mRNA expression of the genes INSR, IRS2, PIK3R1, PIK3CB, AKT2, and SREBP-1c was measured at 4 or 12 weeks, normalised to the housekeeping gene ATCB. Four groups of cross-fostered offspring existed: offspring exposed to a chow diet during pregnancy cross-fostered to a chow fed dam during lactation (CC, *n* 8) or a cafeteria fed dam (CO, *n* 7-8), offspring exposed to a cafeteria diet during pregnancy cross-fostered to a chow fed dam during lactation (OC, *n* 7-8) or a cafeteria fed dam (OO, *n* 5-6).

Age	Group							
	CC		CO		OC		OO	
	Mean	SEM	Mean	SEM	Mean	SEM	Mean	SEM
**4 weeks**								
INSR/ATCB	1.06	0.14	0.96	0.21	1.01	0.31	1.34	0.31
IRS2/ATCB	0.96	0.16	0.93	0.15	1.12	0.21	1.31	0.18
PIK3R1/ATCB	1.14	0.16	1.02	0.17	1.09	0.21	1.37	0.26
PIK3CB/ATCB	1.12	0.07	0.94	0.12	1.06	0.16	1.36	0.17
AKT2/ATCB	1.21	0.22	1.31	0.18	1.45	0.25	1.11	0.10
SREBP-1C/ATCB	1.09	0.17	1.04	0.12	1.15	0.22	0.89	0.14
**12 weeks**								
INSR/ATCB	1.06	0.07	1.15	0.08	1.35	0.05	1.18	0.15
IRS2/ATCB	1.40	0.21	1.14	0.12	1.52	0.16	1.55	0.23
PIK3R1/ATCB	0.81	0.05	0.91	0.06	0.89	0.08	0.96	0.07
PIK3CB/ATCB	1.03	0.08	1.16	0.06	*1.30	0.09	1.06	0.11
AKT2/ATCB	0.97	0.09	1.13	0.09	1.14	0.09	1.06	0.21
SREBP-1C/ATCB	1.11	0.27	1.47	0.30	1.23	0.20	0.90	0.19

Supplementary Table S2. Gastrocnemius muscle insulin signalling mRNA expression in male offspring.
Values are for mean and SEM. Male offspring insulin signalling mRNA expression of the genes INSR, IRS2, PIK3R1, PIK3CB, AKT2, and SREBP-1c was measured at 4 or 12 weeks, normalised to the housekeeping gene ATCB. Four groups of cross-fostered offspring existed: offspring exposed to a chow diet during pregnancy cross-fostered to a chow fed dam during lactation (CC, *n* 5-7) or a cafeteria fed dam (CO, *n* 6-8), offspring exposed to a cafeteria diet during gestation cross-fostered to a chow fed dam during lactation (OC, n *5*-7) or a cafeteria fed dam (OO, *n* 5).

* Effect of maternal pregnancy diet and maternal lactation diet (P=0.034).

Age	Group							
	CC		CO		OC		OO	
	Mean	SEM	Mean	SEM	Mean	SEM	Mean	SEM
**4 weeks**								
INSR/ATCB	1.02	0.07	1.05	0.10	1.19	0.12	1.21	0.08
IRS2/ATCB	1.29	0.20	1.47	0.12	1.44	0.19	1.95	0.38
PIK3R1/ATCB	1.05	0.17	1.02	0.13	1.37	0.26	1.10	0.07
PIK3CB/ATCB	1.06	0.15	1.11	0.13	*1.53	0.17	*1.67	0.26
AKT2/ATCB	0.94	0.06	1.03	0.13	*1.31	0.09	*1.14	0.04
SREBP-1C/ATCB	1.01	0.07	1.03	0.16	*1.56	0.22	*1.33	0.09
**12 weeks**								
INSR/ATCB	0.99	0.21	1.33	0.16	1.04	0.11	1.13	0.13
IRS2/ATCB	0.80	0.14	1.00	0.28	1.18	0.14	0.83	0.13
PIK3R1/ATCB	0.91	0.16	0.86	0.17	0.92	0.14	0.79	0.12
PIK3CB/ATCB	0.69	0.11	0.83	0.13	0.81	0.10	1.04	0.19
AKT2/ATCB	0.82	0.19	0.85	0.23	1.06	0.09	0.83	0.21
SREBP-1C/ATCB	0.77	0.17	1.17	0.20	1.31	0.26	1.09	0.18

Supplementary Table S3. Perirenal adipose tissue insulin signalling mRNA expression in male offspring.
Values are for mean and SEM. Male offspring insulin signalling mRNA expression of the genes INSR, IRS2, PIK3R1, PIK3CB, AKT2, and SREBP-1c was measured at 4 or 12 weeks, normalised to the housekeeping gene ATCB. Four groups of cross-fostered offspring existed: offspring exposed to a chow diet during pregnancy cross-fostered to a chow fed dam during lactation (CC, *n* 5-7) or a cafeteria fed dam (CO, *n* 6-7), offspring exposed to a cafeteria diet during gestation cross-fostered to a chow fed dam during lactation (OC, *n* 5-7) or a cafeteria fed dam (OO, *n* 4-5).
* Effect of maternal pregnancy diet ($P<0.05$).

Age	Group							
	CC		CO		OC		OO	
	Mean	SEM	Mean	SEM	Mean	SEM	Mean	SEM
**4 weeks**								
INSR/ATCB	0.93	0.04	0.95	0.06	0.99	0.05	1.00	0.07
IRS2/ATCB	1.24	0.12	1.35	0.13	1.21	0.18	1.15	0.11
PIK3R1/ATCB	0.77	0.07	0.73	0.03	0.87	0.09	0.75	0.07
PIK3CB/ATCB	1.08	0.03	1.05	0.03	1.10	0.05	1.08	0.05
AKT2/ATCB	1.62	0.09	1.41	0.13	1.76	0.18	1.53	0.10
SREBP-1C/ATCB	1.56	0.09	1.59	0.27	1.89	0.18	1.62	0.20
**12 weeks**								
INSR/ATCB	0.83	0.03	0.82	0.07	0.78	0.07	0.92	0.13
IRS2/ATCB	1.03	0.04	0.77	0.12	0.93	0.12	0.87	0.16
PIK3R1/ATCB	0.83	0.07	0.67	0.04	0.73	0.08	0.64	0.08
PIK3CB/ATCB	0.92	0.01	0.92	0.02	0.93	0.05	0.97	0.10
AKT2/ATCB	0.98	0.04	1.09	0.05	0.95	0.06	0.99	0.10
SREBP-1C/ATCB	0.78	0.10	0.76	0.05	0.74	0.14	0.84	0.09

Supplementary Table S4. Gonadal adipose tissue insulin signalling mRNA expression in male offspring.
Values are for mean and SEM. Male offspring insulin signalling mRNA expression of the genes INSR, IRS2, PIK3R1, PIK3CB, AKT2, and SREBP-1c was measured at 4 or 12 weeks, normalised to the housekeeping gene ATCB. Four groups of cross-fostered offspring existed: offspring exposed to a chow diet during pregnancy cross-fostered to a chow fed dam during lactation (CC, *n* 6-8) or a cafeteria fed dam (CO, *n* 6-7), offspring exposed to a cafeteria diet during gestation cross-fostered to a chow fed dam during lactation (OC, *n* 4-8) or a cafeteria fed dam (OO, *n* 4-6).
